# Prevalence and risk factors of schistosomiasis and soil-transmitted helminthiases among preschool aged children (1–5 years) in rural KwaZulu-Natal, South Africa: a cross-sectional study

**DOI:** 10.1186/s40249-019-0561-5

**Published:** 2019-06-16

**Authors:** Hlengiwe Sacolo-Gwebu, Moses Chimbari, Chester Kalinda

**Affiliations:** 1 School of Nursing and Public Health, College of Health Sciences, University of KwaZulu-Natal, Howard Campus, Durban, South Africa; 2 University of Namibia, Katima Mulilo Campus, Winela Road, Box 1096, Katima Mulilo, Namibia

**Keywords:** Prevalence, Risk factor, Schistosomiasis, Soil-transmitted helminth, Preschool aged children, KwaZulu-Natal, South Africa

## Abstract

**Background:**

Despite efforts to control neglected tropical diseases (NTDs), schistosomiasis and soil-transmitted helminthiases remain widely prevalent in sub-Saharan Africa. Recent data suggest that these infections are prevalent among preschool aged children (PSAC) in poor communities. Evidence of schistosomiasis and soil-transmitted helminths (STH) infection patterns and prevalence among PSAC is essential for effective treatment and control programmes. The aim of the study was to determine the prevalence, intensity and risk factors of schistosomiasis and STH infection among PSAC in the Ingwavuma area of uMkhanyakude District, South Africa.

**Methods:**

A cross-sectional study was conducted among 1143 PSAC aged 1–5 years in 34 preschools and early childhood development (ECD) centres. Data on risk factors was collected using a semi-structured questionnaire. A Kruskal–Wallis test was used to compare the differences in infection intensity with age. Pearson Chi-square test and multivariate logistic regression were performed to assess the association between PSAC infection status, sociodemographic, household, water and sanitation variables and hygiene practices of PSAC and their caregivers.

**Results:**

We observed a low prevalence of *Schistosoma haematobium* (1.0%) and *S. mansoni* (0.9%). The prevalence of *Ascaris lumbricoides* (18.3%) was high compared to *Trichuris trichiura* (1.2%), hookworms (1.6%) and *Taenia* (6.4%). The odds of schistosome infection were lowest among PSAC under younger (15–24 years) caregivers (0.1, 95% *CI*: 0.02–0.54) and those who used tap water (0.3, 95% *CI*: 0.09–0.78) for domestic purposes. Schistosome infection was however higher among PSAC who bathed in river water (17.4, 95% *CI*: 5.96–51.04). STH infection on the other hand was lowest among PSAC who did not play in soil (0.1, 95% *CI*: 0.51–0.28), were from households that used tap water for domestic purposes (0.5, 95% *CI*: 0.27–0.80) and PSAC under the care of younger (25–35 years) caregivers (0.3, 95% *CI*: 0.10–0.75). The risk of STH infection was highest among PSAC who did not wash their hands with soap (3.5, 95% *CI*: 1.04–11.67) and PSAC whose nails were not trimmed (3.6, 95% *CI*: 1.75–7.26).

**Conclusions:**

The findings show low prevalence and infection intensity of schistosomiasis and STH infection except *A. lumbricoides* among PSAC. Factors predicting schistosomiasis and STH infection among PSAC were related to caregivers’ age, educational status, water and hygiene practices. STH infection was exclusively associated with PSAC playing and handwashing habits. These findings highlight the need to include PSAC caregivers in schistosomiasis and STH prevention and control programmes.

**Electronic supplementary material:**

The online version of this article (10.1186/s40249-019-0561-5) contains supplementary material, which is available to authorized users.

## Multilingual abstracts

Please see Additional file 1 for translations of the abstract into the five official working languages of the United Nations.

## Background

Schistosomiasis, also known as bilharzia and soil-transmitted helminths (STH, *Ascaris lumbricoides* [roundworms], *Trichuris trichiura* [whipworms], and hookworms) are the most prevalent parasitic infections worldwide [1]. Global estimates suggest that schistosomes infect about 230 million people while *A. lumbricoides, T. trichuris* and hookworms infect 1.221 billion, 795 million and 740 million people, respectively [2]. Ninety per cent of the people infected worldwide reside in sub-Saharan Africa (SSA). Due to the overlap in their geographical distributions [3], schistosomiasis and soil-transmitted helminthiases can occur independently or jointly depending on the nature of risk factors and host related attributes [1–4]. Risk factors such as access to safe water and sanitary related practices of caregivers and children have been reported to be associated with STH infection [5, 6]. Whilst school aged children (SAC) acquire schistosomiasis by participating in risky water practices [7, 8], preschool aged children (PSAC) are passively exposed to infection when their parents perform daily water-related chores [9, 10].

PSAC may be exposed to infection within the first 5 years of life and thus contribute to the observed heavy burden of infection in the SAC. The burden associated with schistosomiasis and STH infection exhibits notable commonalities; (i) both infections thrive in poverty-stricken areas with limited or no access to safe water supply and basic sanitation [3, 11], (ii) pathology is related to worm burden and may lead to death in heavily infected individuals, (iii) they retard the physical and cognitive development of children and hinder educational advancement [12–14], (iv) schistosomes and STH-immunosuppressive features may predispose infected individuals to HIV/AIDS [15–17], and (v) chronic infection may cause severe illness and irreversible disabilities such as cancers of the bladder, prostate, kidney, liver or intestines [18–20] .

In South Africa, schistosomiasis and STH infection are more prevalent amongst disadvantaged children who live in poor rural communities. High levels of infection have been documented amongst children in most provinces in South Africa, including KwaZulu-Natal [7, 21], Mpumalanga [22], Port Elizabeth [23] and Limpopo [24]. South Africa is a signatory to the World Health Assembly (WHA) resolution 54.19 [25], which calls for regular treatment of worms in high-risk groups. Mass drug administration for the treatment of soil-transmitted helminthiases is currently rolled out in primary schools. However, the mass treatment for schistosomiasis treatment has not yet been initiated. In South Africa about 5.2 million people need annual preventive chemotherapy against schistosomiasis per year, mostly targeting SAC [26]. Schistosomiasis and soil-transmitted helminthiases control measures include the provision of water and sanitation, health education, snail control and treatment [27, 28]. Currently, control programmes mainly focus on the treatment aspect and yet studies have shown that treatment-based control programmes have a temporal effect on transmission [29]. It is for this reason that the World Health Organization (WHO) expects endemic countries to develop comprehensive strategies aiming at eliminating soil-transmitted helminthiases and schistosomiasis amongst other NTDs by 2020 [3, 27]. This move demands urgent and aggressive programmes designed to both control morbidity and curtail the spread of infection. Endemic countries are still grappling with the search for the most important factors responsible for the rapid spread of soil-transmitted helminthiases and schistosomiasis. The knowledge of contextual risk factors may assist programme managers in channelling resources where it matters. Our study is thus timely and pertinent considering the shift from morbidity control to elimination of schistosomiasis and soil-transmitted helminthiases in many endemic countries. The objective of this study was to determine the prevalence and risk factors of schistosomiasis and STH infection among PSAC aged 1–5 years in the Ingwavuma area of uMkhanyakude District, South Africa.

## Methods

### Study area and population

**Fig. 1:**
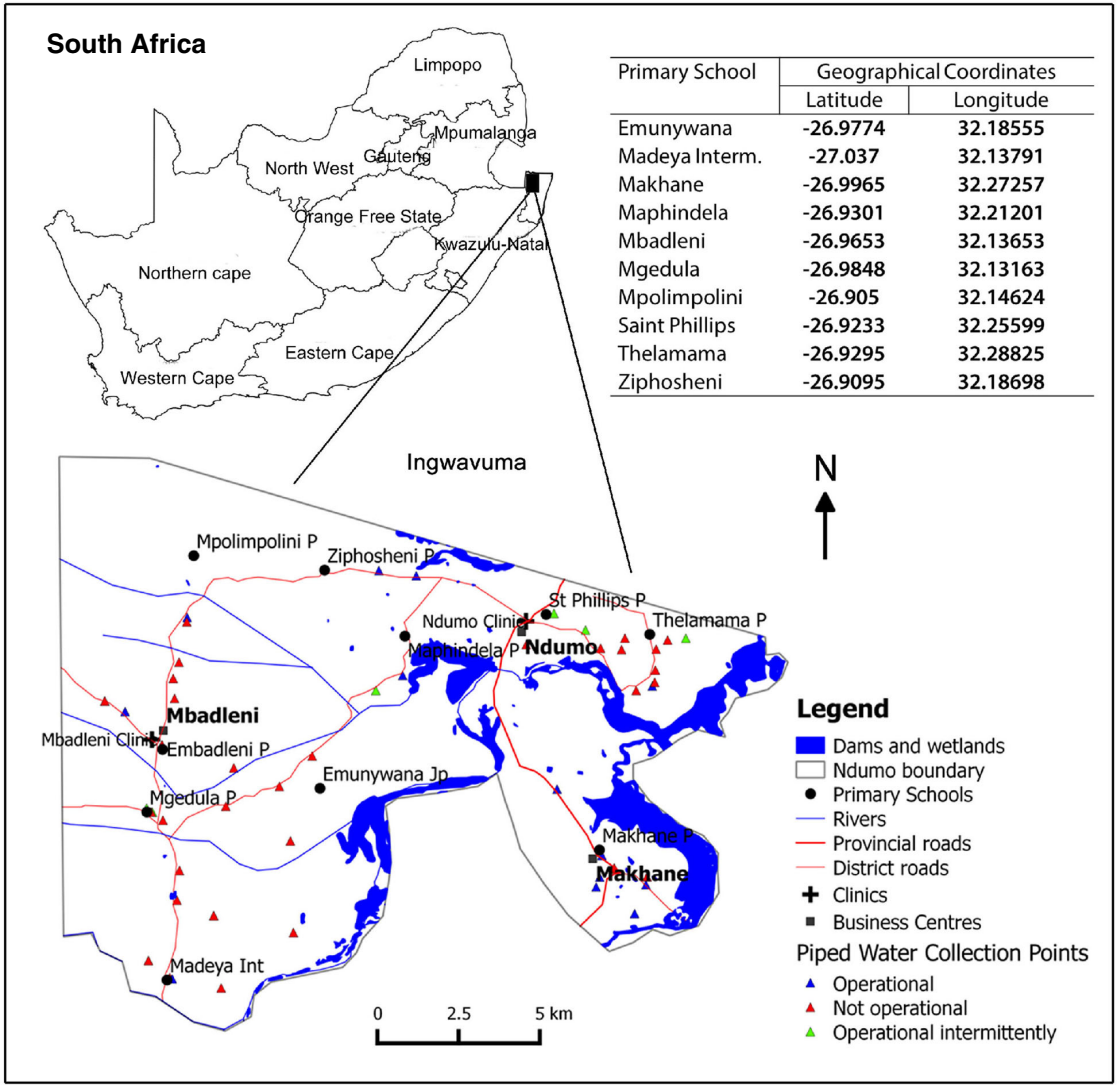
Map of Ingwavuma area of Jozini Municipality in uMkhanyakude District, KwaZulu-Natal, South Africa

This study was conducted in Ingwavuma rural community located in the northern part of uMkhanyakude (see Fig. 1, source: Manyangadze, Chimbari [30]), the third-poorest district in KwaZulu-Natal, South Africa [31]. Ingwavuma area, our study site is located within the Jozini local municipality which covers about 32% (3057 km^2^) of uMkhanyakude District [32]. Jozini has a total population of 186 502 people of which 89% reside in the rural areas. Most of the population (99.2%) are black Africans of which 94.6% are Zulu speakers. The Jozini municipality has a total of 20 wards. Our study was carried out in three wards covering 582 km^2^ and a population of 28 384 people [32]. The unemployment rate in the district is 72.1%. People in this area live under the jurisdiction of traditional leadership and have limited access to basic services. For instance, 60% of the population lacks access to electricity, 38.2% have no access to tap water, whilst 18.2% have no access to toilets [31].

The area is arid with seasonal dams and characterized by hot and humid summer with a cooler and drier winter season. Multiple hills within the area improve water drainage, the Pongola and Ingwavuma rivers serve as the major drainage channels and are major sources of irrigation water [33]. Except for a minority of households with access to tapped water, most households rely on rivers, seasonal streams, dams and ponds for gardening and laundry. However, drought conditions in the study area from 2014 to 2017 dried out most water bodies [34]. uMkhanyakude District is formed of the following three related vegetation types: (i) mixed compound leaved short woodland (trees between 5 and 10 m) and wooded grasslands; (ii) open bushveld with dominant *Acacia* and *Combretum* species and *Themeda triandra* grass being the dominant grass in undisturbed areas; and (iii) open, tall, sour, wiry grasslands, often dotted with low bushes and solitary savannah trees. Most of the area is covered by sandy soil, particularly in the Maputaland region. The rest of the area is covered by Namib soil which is deep, acidic and well-drained; however, clay soil is found in some few areas [35, 36].

### Study design and enrolment of participants

**Fig. 2:**
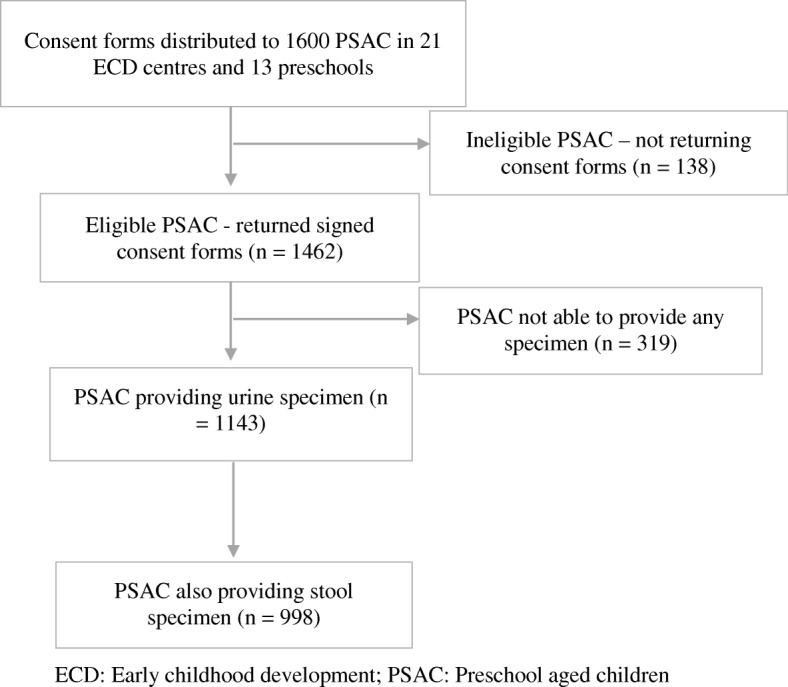
Sampling methodology

This was a cross-sectional study in which 1143 PSAC participated (see Fig. 2). Prevalence of schistosomiasis and STH infection among PSAC in the study area is unknown. This study was part of a bigger ongoing study that sought to screen all PSAC in accessible early child development (ECD) centres and pre-schools in the area whose caregivers consented to the study. Consent forms were distributed to 1600 PSAC but only 1462 returned signed consent forms and were eligible to participate in the study. The participants were drawn from 21 ECD centres and 13 preschools from 14 main villages within the Ingwavuma area. In the South African public education system, preschools are commonly known as grade R or grade 0 and are found within primary schools whilst ECD centres mostly standalone. Therefore, all primary schools within wards 15, 16 and 17 in our study area were eligible for inclusion in the study and were therefore selected. Furthermore, registered ECD centres around the selected primary schools were included in the study. During sample collection visits, 319 children could not provide any specimen and were therefore excluded from the study. Our study sample comprised of 1143 PSAC who provided urine samples out of whom 998 provided stool samples. Caregivers (parents and guardians) of children enrolled in the study responded to a schistosomiasis and soil-transmitted helminthiases risk factor questionnaire. Caregivers were eligible to participate if (1) they were present in schools and ECD centres during the day of screening, (2) their children had provided urine and stool specimens; and (3) were willing to participate.

### Data collection

#### Urine and stool sample collection

The study was conducted from June to September 2018. Urine and stool samples were collected using baby potties. Samples were collected between 10:00 am and 2:00 pm for optimal egg count necessary for the diagnosis of schistosomiasis and STH infection [37]. Caregivers of PSAC were oriented on specimen collection procedures prior to specimen collection. Potties and specimen bottles were marked with identification codes representing each child and distributed to project research assistants and caregivers of PSAC. The identification codes on specimen bottles were matched with caregiver questionnaire identification codes such that a caregiver shared the same code with his or her child. Collected samples were enclosed in the coded specimen bottles, covered in wooden boxes placed in an area with shade to maintain sample quality and immediately transported to a processing site in the study area. Distant schools were 30 min away from the processing site and most samples were processed within two hours of collection.

Urine samples were processed using the filtration technique [38] whilst stool samples were processed using the Kato-Katz technique [39]. For the filtration, we used nucleopore membrane filters with a diameter 13 mm and pore size 12 μm. Each urine sample was well mixed, and 10 ml was filtered through the membrane filter which was then placed on a microscope slide. A drop of Lugol’s iodine was added and the slide was examined under a microscope using objective × 10 and × 40 by two experienced laboratory technicians. The number of *S. haematobium* eggs per 10 ml were counted and recorded for each child [37]. For the Kato-Katz technique, each stool sample was pressed through a sieve to remove large particles. Part of the sieved stool was then transferred to the hole of a template on a slide. The hole was filled after which the template was removed and the remaining sieved sample (10 mg) was covered with cellophane which had been pre-soaked in glycerol. The preparation was pressed with a cover slip or a slide to become flat. The slide was then viewed, and eggs were counted, and the number of eggs per gram of stool (EPG) was calculated [37, 40]. Each slide was read independently by two technicians. *S. haematobium* infection was determined by the presence of eggs in 10 ml of urine while *S. mansoni*, and STH infection were determined by the presence of eggs in 1 g of stool.

The intensity of infection was classified into the three levels defined by WHO as light, moderate and heavy infection. The classification of thresholds on infection intensity varied between parasites; for *S. haematobuim,* < 50 eggs/10 ml was classified as light intensity, and > 50 eggs/10 ml was heavy intensity; for *S. mansoni,* < 100 EPG was light intensity, 100–400 EPG was moderate intensity, and > 400 EPG was heavy intensity [37]. For *A. lumbricoides,* 1–4999 EPG was light intensity, 5000–49 999 EPG was moderate intensity, and > 50 000 EPG was heavy intensity; for *T. trichiura,* 1–999 EPG was light intensity, 1000–9999 EPG was moderate intensity, and > 10 000 EPG was heavy intensity; for hookworm, 1–1999 EPG was low intensity, 2000–3999 EPG was moderate intensity, and > 4000 EPG was heavy intensity [37]. Unlike STH and schistosomiasis, it is not easy to determine the intensity of *Taenia* thus we only determined the prevalence of *Taenia* species. The difficulty is related to the fact that *Taenia* eggs are expelled in gravid proglottids thus it is difficult to count individual eggs. Each detached proglottid segment is estimated to contain about 50 000–60 000 fertile eggs [41–43].

### Data analysis

Data were analyzed using SPSS version 25.0 computer software (IBM Corporation, New York, USA) after checking its completeness. The dependent variables were schistosomiasis and STH infection status among PSAC (where STH infection was taken to mean infection with *A. lumbricoides, T. trichiura*, and hookworm) and the intensity of infection. The prevalence and arithmetic mean intensity of infection among all children was examined with 95% confidence intervals (*CI*s). In estimating the prevalence and mean intensity of infection, children were stratified according to gender. Non-parametric one-way ANOVA (Kruskal–Wallis) was used to compare the differences in intensity of infection with age. Furthermore, Pearson Chi-square test was used to determine the differences in prevalence. Statistically significant variables in the bivariate analysis were used as predictors in the multivariate logistic regression. This was done for data relating to both children and caregiver to measure the strength of the association between the risk factors and infection status. The results were expressed as odds ratio (*OR*) with their 95% *CI* and statistical significance level, *P* < 0.05. Potential risk factors explored were demographic factors (sex, age, occupation and education level), water and sanitary related factors and behavioural factors associated with schistosomiasis and STH infection.

## Results

### Sample description

Overall, 1462 children were enrolled in the study but only 1143 gave urine samples of which 998 also gave stool samples. The sample comprised of 557 (48.7%) males and 586 (51.3%) females. The mean age of PSAC involved in the study was 3.94 (± 1.10 years) and their ages ranged between 1 and 5 years. The sociodemographic characteristics of the study participants are in Table 1.

**Table 1:** Sociodemographic characteristics of preschool aged children and caregivers

Variables	Character	Frequency Proportion (%)	
Gender of PSAC (*n* = 1143)	Female	586	51.3
	Male	557	48.7
Age in years of PSAC (*n* = 1143)	1	35	3.1
	2	104	9.1
	3	203	17.7
	4	348	30.4
	5	453	39.6
Age in years of caregivers (*n* = 442)	15–24 years	105	23.5
	25–34 years	174	38.0
	35–44 years	85	19.0
	45–54 years	44	11.8
	> 55 years	34	7.7
Marital status of caregivers (*n* = 442)	Single - never married	364	82.4
	Divorced	22	5.0
	Widowed	14	3.2
	Married	42	9.5
Educational status of caregivers (*n* = 442)	No formal education	65	14.7
	Primary school	103	23.3
	Secondary school	258	58.4
	College level	16	3.6
Occupation of caregiver (*n* = 442)	Not working	372	84.2
	Self-employed	39	8.8
	Employed	31	7.0

### Prevalence of schistosomiasis and soil-transmitted helminthiases

Of the 1143 PSAC who provided urine samples, 11 (0.1%) tested positive for *S. haematobium*. Of these, three cases were males while eight cases were females. On the other hand, of 998 PSAC who provided stool samples nine (0.9%) cases were *S. mansoni* infection; seven males and two females. *A. lumbricoides*, *T. trichiura*, hookworm and *Taenia* were diagnosed in the stool specimens analyzed. *A. lumbricoides* was the predominant intestinal helminth infection with a prevalence of 18.3% (95% *CI*: 13.9–18.3%) and the lowest STH infection was for *T. trichiura* which had a prevalence of 1.2% (95% *CI*: 0.54–1.83%) (Table 2).

**Table 2:** Prevalence of schistosomiasis and soil-transmitted helminthiases among preschool aged children by age

Age group	Frequency	*S. haematobium* (95% *CI*)	*S. mansoni* (95% *CI*)	*A. lumbricoides* (95% *CI*)	*T. trichuris* (95% *CI*)	Hookworm (95% *CI*)	*Taenia* species (95% *CI*)
1-year-old	35	0	0	11.4% (4.41–25.31%)	0	0	0
2-year-old	104	0	0	6.7% (1.89–11.51%)	0	1.0% (0.17–5.24%)	2.9% (0.98–8.13%)
3-year-old	203	1.0% (0.27–3.53%)	0	10.3% (6.86–15.29%)	0.5% (0.09–2.73%)	0.1% (0.27–3.53%)	3.4% (1.68–6.95%)
4-year-old	348	0.9% (0.00–1.89%)	1.7% (0.34–3.06%)	21.3% (17.29–25.86%)	1.4% (0.62–3.32%)	2.3% (1.17–4.47%)	7.2% (4.91–10.39%)
5-year-old	453	1.3% (0.61–2.87%)	0.7% (0.22–1.93%)	17.0% (13.86–20.78%)	1.3% (0.61–2.87%)	1.1% (0.48–2.57%)	6.4% (4.15–8.65%)
Total	1143						
*χ*^*2*^ (*P*-value)		2.00 (0.730)	6.75 (0.151)	19.65 (0.001)	2.92 (0.570)	1.87 (0.520)	7.53 (0.108)

Note: Except where indicated as *P*-value, figures in brackets show 95% confidence intervals

Analysis of our data showed that the prevalence of *A. lumbricoides* increased with age (*P* < 0.001). Furthermore, the prevalence of *Taenia* was high among males (*P* < 0.012). There was no statistically significant difference between males and females in relation to other helminth infections (*P > 0.05*) (Table 3).

**Table 3:** Prevalence of schistosomiasis and soil-transmitted helminthiases among preschool aged children by gender

Gender	Frequency	*S. haematobium* (95% *CI*)	*S. mansoni* (95% *CI*)	*A. lumbricoides* (95% *CI*)	*T. trichuris* (95% *CI*)	Hookworm (95% *CI*)	*Taenia* species (95% *CI*)
Female	586	0.3%(0.00–0.74%)	0.3% (0.00–0.74%)	14.2% (11.64–17.31%)	0.7% (0.26–1.74%)	1.0% (0.47–0.23%)	4.0% (2.63–5.81%)
Male	557	1.3% (0.18–1.57%)	1.3% (0.61–2.58%)	18.0% (14.99–21.35%)	1.4% (0.73–2.81%)	1.8% (0.98–3.28%)	7.4% (2.63–5.81%)
Total	1143	1.0% (0.54–1.71%)	0.8% (0.42–1.49%)	16.0% (13.87–18.13%)	1.0% (0.42–1.58%)	1.4% (0.86–2.26%)	5.6% (4.51–9.07%)
*χ*^*2*^ (*P*-value)		2.05 (0.152)	3.06 (0.080)	3.05 (0.081)	1.56 (0.211)	1.23 (0.267)	6.38 (0.012)

Note: Except where indicated as *P*-value, figures in brackets show 95% confidence intervals

### Coinfection and intensity of schistosomiasis and soil-transmitted helminthiases among PSAC

The arithmetic mean of infection intensity for *S. haematobium* was 30.40 eggs/10 ml (95% *CI*: 27.30–33.80) and it was 6.90 EPG (95% *CI*: 5.47–8.61) for *S. mansoni*. There were no significant differences in the intensity of *S. haematobium* (*χ*^*2*^ = 1.512, *P* = 0.824) and *S. mansoni* (*χ*^*2*^ = 0.122, *P* = 0.998) infection with age. Significant differences were observed in the number of infections per child with age (*χ*^*2*^ = 13.35, *P* = 0.0097) showing that the number of species harboured increased with age. The mean intensity of infection for *A. lumbricoides* was 2.17 EPG (95% *CI*: 1.74–2.61) while it was 0.56 EPG (95% *CI*: 0.369–0.747) for *T. trichiuris.* The prevalence of both mono and poly-infections increased with age, poly-infection was highest (27.8%) amongst 4-year-olds and least (0%) among 1-year-olds (Table 4). However, no significant difference was observed between poly infection status and gender (*χ*^*2*^ = 2.59, *P* = 0.107).

**Table 4:** Number of infections harboured by preschool aged children by age

Age group	Infections status		
	No infection, *n*	Mono-infection, *n*	Poly-infection, *n* (%^a^)
1-year-old	31	4	0 (0)
2-year-old	94	9	1 (10.0)
3-year-old	176	22	5 (18.2)
4-year-old	251	70	27 (27.8)
5-year-old	344	90	19 (17.4)

^a^Number in brackets is poly-infection rate expressed as a percentage of poly-infections within infected PSAC

### Risk factors associated with schistosomiasis in PSAC

In both bivariate and multivariate analysis, the caregiver’s age group, caregivers’ habit of bathing children in river water and source of domestic water supply were significantly associated with schistosome infection. The odds of schistosome infection were lower among PSAC with younger (15–24 years) caregivers (0.1, 95% *CI*: 0.02–0.54) and those who used tap water (0.3, 95% *CI*: 0.09–0.78) for domestic purposes. Schistosome infection was however higher among PSAC whose caregivers reported to be bathing them in river water (17.4, 95% *CI*: 5.96–51.04). (Table 5).

**Table 5:** Bivariate and multivariate analysis of schistosomiasis across selected factors among preschool aged children

Variable		Schistosome infection			Chi-square test	Multivariate logistic regression
		Positive	Negative	Total (*n* = 442)	*P*-value	aOR (95% CI)
Children’s gender	Male	10	222	232	0.990	–
	Female	9	201	210		
Children’s age	1-year-old	0	21	21	0.225	–
	2-year-old	0	36	36		
	3-year-old	2	74	76		
	4-year-old	9	120	129		
	5-year-old	8	172	180		
Caregivers’ age group	15–24 years	2	103	105	0.001	0.09 (0.02–0.54) ^a^
	25–34 years	4	170	174		0.11 (0.03–0.49) ^a^
	35–44 years	3	82	85		0.20 (0.04–0.99) ^a^
	45–54 years	3	41	44		0.31 (0.06–1.57)
	> 55 years	7	27	34		1
Caregivers’ education	No formal education	4	61	65	0.119	–
	Primary school	8	95	103		
	Secondary school	7	251	258		
	College level	0	16	16		
Caregivers’ occupation	Not working	16	356	372	0.728	–
	Self employed	1	38	39		
	Employed	2	29	31		
Main source of domestic household water	Tap water	7	288	295	0.005	0.26 (0.09–0.78) ^a^
	Open source water	12	135	147		1
How often does your child swim in the river	Never	11	296	307	0.100	–
	Sometimes	2	69	71		
	Regularly	3	19	22		
	All the time	3	39	42		
Do you sometimes bath your child in river water	Yes	11	30	41	0.001	17.44 (5.96–51.04) ^b^
	No	8	393	401		1
How often does your child accompany you when performing daily chores	Never	9	264	264	0.563	–
	Sometimes	4	91	91		
	Regularly	3	39	39		
	All the time	3	48	48		
How often do you treat fresh river water meant for bathing your child	Never	9	197	206	0.999	–
	Sometimes	4	88	92		
	Regularly	2	43	45		
	All the time	4	95	99		

^a^ a*OR P*-value < 0.05; ^b^ a*OR P*-value < 0.01

### Risk factors associated with soil-transmitted helminthiases in PSAC

The prevalence of STH infection was significantly associated (*P* < 0.05) with the following factors: marital status, educational status, caregiver’s age group, type of toilet used at home, main source of domestic water, frequency of trimming child’s fingernails, caregiver’s handwashing habit after visiting the toilet, child’s habit of washing hands with soap and water and child’s habit of playing in soil (Table 6). In a multivariate logistic regression results presented in Table 5, the odds of STH infection were lowest among PSAC who did not play in soil (0.12, 95% *CI*: 0.51–0.28), those from households that used tap water for domestic purposes (0.47, 95% *CI*: 0.27–0.80) and PSAC under the care of caregivers within the age group 25–35 years (0.27, 95% *CI*: 0.10–0.75). The risk of STH infection was higher among PSAC who did not wash their hands with soap and water (3.49, 95% *CI*: 1.04–11.67), PSAC with primary level educated caregivers (4.16, 95% *CI*: 0.49–35.63) and those who did not have their nails trimmed (3.56, 95% *CI*: 1.75–7.26).

**Table 6:** Bivariate and multivariate analysis of soil-transmitted helminthiases in relation to selected factors among preschool aged children

Variable		Soil-transmitted helminths infection			Chi-square test	Multivariate logistic regression
		Positive	Negative	Total (*n* = 442)	*P*-value	a*OR* (95% *CI*)
Children’s gender	Male	75	158	233	0.070	–
	Female	51	158	209		
Children’s age	1-year-old	7	14	21	0.972	–
	2-year-old	11	27	38		
	3-year-old	20	57	77		
	4-year-old	37	90	127		
	5-year-old	51	128	179		
Care givers’ educational status	No formal education	21	44	65	0.014	2.05 (0.21–20.01)
	Primary school	20	83	103		4.16 (0.49–35.63)
	Secondary school	84	174	258		1.69 (0.18–15.67)
	College level	1	15	16		1
Caregivers’ occupational status	Not working	105	356	372	0.891	–
	Self employed	11	38	39		
	Employed	10	29	31		
Caregivers’ age group	15–24 years	25	80	105	0.001	0.28 (0.09–0.85) ^b^
	25–34 years	37	137	174		0.27 (0.10–0.75) ^b^
	35–44 years	30	55	85		0.53 (0.19–1.47)
	45–54 years	15	29	44		0.63 (0.21–1.95)
	> 55 years	19	15	34		1
Do you have a toilet at home	Yes	121	312	433	0.069	–
	No	5	4	9		
Main source of domestic household water	Tap water	68	227	295	0.006	0.47 (0.27–0.80) ^b^
	Open source water	58	89	147		1
How often do you wash your hands after visiting the toilet	Never	28	36	64	0.017	4.45 (0.20–1.14)
	Sometimes	77	185	262		3.52 (0.04–5.43)
	Regularly	11	43	54		1.85 (0.10–0.58) ^a^
	All the time	10	52	62		1
How often do you trim your child’s finger nails	Never	59	86	145	0.001	3.56 (1.75–7.26) ^b^
	Sometimes	33	73	106		2.48 (1.15–5.33) ^a^
	Regularly	16	68	84		1.23 (0.50–5.33)
	All the time	18	89	107		1
How often do you use soap and water when washing hands	Never	9	25	34	0.722	–
	Sometimes	39	87	126		
	Regularly	12	41	53		
	All the time	66	162	228		
What does your child normally use to wash hands	Water only	108	274	382	0.783	–
	Soap and water	18	42	60		
How often does your child wash his or her hands using soap and water	Never	67	124	191	0.005	3.49 (1.04–11.67) ^b^
	Sometimes	46	115	161		4.22 (1.23–14.53) ^b^
	Regularly	9	50	59		1.67 (0.40–7.10)
	All the time	4	27	31		1
How often does your child play with soil	Never	11	92	103	0.001	0.12 (0.51–0.28) ^a^
	Sometimes	24	73	97		0.44 (0.21–0.92) ^a^
	Regularly	47	100	147		0.43 (0.23–0.82) ^a^
	All the time	44	51	95		1
How often does your child bite his or her nails	Never	2	13	15	0.056	–
	Sometimes	32	114	146		
	Regularly	36	68	104		
	All the time	56	121	177		
How do you treat water meant for drinking	Boiling	23	50	73	0.711	–
	Filtration	2	8	10		
	Use chemicals	7	25	32		
	Nothing	94	233	327		

^a^ a*OR P*-value < 0.05; ^b^ a*OR P*-value < 0.01

## Discussion

This study examined the prevalence, intensity and risk factors of schistosomiasis and soil-transmitted helminthiases among PSAC aged 1–5 years. To the best of our knowledge, this is the first study in South Africa that has attempted to examine schistosomiasis and soil-transmitted helminthiases among PSAC. Previous studies in South Africa have focused on schistosomiasis and or STH infection among SAC where the reported prevalence ranged from 24 to 80% [7, 16, 21, 22, 44, 45]. In our study, the prevalence of schistosomiasis was low. Our findings are similar to those for a study in Tanzania [46] that reported a 1.9% prevalence of *S. haematobium* among 424 PSAC who all had light infection. In contrast to our study, studies conducted in Mali [47], Zimbabwe [48], Uganda [49] and Nigeria [9, 10] reported a higher prevalence of schistosomiasis ranging between 21 and 86%. The relatively lower prevalence of schistosomiasis in our study may be related to the drought that affected uMkhanyakude, Ingwavuma area from 2014 to 2016 [34]. Rainfall patterns changed considerably in the study area leaving most transmission sites dry. This also resulted in a decline in the prevalence of schistosomiasis among SAC within the study area [34]. In addition, the administration of praziquantel to SAC within the study area since 2015 [50] may have led to a reduction in infection rates. There is evidence that treating SAC infected with schistosomiasis tends to lower the prevalence among the entire population [51, 52].

In our study, PSAC gender and age were not significant predictors of schistosome infection. These findings are consistent with studies conducted in Tanzania [46], Nigeria [53] and Ethiopia [54]. Furthermore, findings from the study done in Nigeria [53] also suggest that the gender and age of PSAC may not determine infection status among PSAC since schistosomiasis at this age is believed to be passively acquired depending on the caregiver’s exposure to open water sources. Nevertheless, the study showed that the risk of *A. lumbricoides* infection increased with age among the PSAC. The presence of poly-infection also increased with age of PSAC. This may suggest that those children acquire such infection as they become more independent and adventurous. Our findings corroborate with previous studies that showed age to be a predictor of STH infection among PSAC [53]. The overall prevalence of STH infection was 20.6% with *A. lumbricoides* being the predominant parasite with a prevalence of 18.34%. *A. lumbricoides* infection was significantly associated with the age of the child but not with gender. According to the WHO classification of intensity, all infected PSAC had low infection intensity.

In our study, the odds of infection were higher among PSAC under the care of older caregivers. In rural South Africa, it is common for grandmothers to raise up to two generations of grandchildren [55]. Older caregivers may not be able to actively and closely monitor infants relative to their younger counterparts thus potentially exposing children to activities with high risk of STH infection. In addition, the use of open water sources increased the odds of both schistosomiasis and STH infection. The use of safe water and improved sanitation are essential in preventing the re-emergence of helminth infections after successful treatment. However, such are rarely present in poverty-stricken rural communities [14, 24]. While improved sanitation is central in the control of soil-transmitted helminthiases, it may not be the case in the control of schistosomiasis [21]. If transmission sites remain full of parasite infested water, the prevalence of schistosomiasis is likely to persist regardless of improvements in sanitation or hygiene practices [34]. Literature suggests that behavioural modification strategies such as the provision and promotion of safe water usage, and hygiene practices are key in the control of schistosomiasis and STH transmission [11]. Other researchers, however, stress the importance of integrating control strategies in order to enhance their effectiveness. For instance, behavioural change interventions may not be effective if water, sanitation and hygiene interventions are overlooked [56].

Schistosome infection was associated with water contact activities such as bathing PSAC in the river. Previous studies have shown that *Schistosoma* infection is more prevalent among PSAC whose parents/guardians have high contact with open water sources [10]. In our study, predictors of STH infection were caregivers’ level of education, caregiver’s hand washing habit after toilet use and PSAC habit of washing hands with soap and water. Whilst treatment programmes are necessary to rapidly reduce the burden and morbidity of helminth infections, they are an unsustainable strategy for helminth control and for achieving control and elimination targets [3, 24, 28]. Progress towards achieving the elimination of schistosomiasis and soil-transmitted helminthiases crucially depends on sustainable resolutions that move beyond treating symptoms to reducing exposure. The prevention of reinfection is said to provide sustainable gains for schistosomiasis and soil-transmitted helminthiases control and elimination.

### Limitations

In our study, PSAC only provided single urine and stool samples from which schistosomiasis and STH infection were detected. The WHO encourages the collection of three stool/urine samples per child for accurate detection of infection. The collection of multiple samples also enables the assessment of intra and inter specimen variation of the egg output [57]. Even though researchers were aware of these facts, the difficulty in obtaining multiple samples from children aged 1–5 years limited the implementation of this recommendation. We are also cognizant of the fact that in areas where the prevalence of soil-transmitted helminthiases is low, more sensitive techniques are required [58]. While our findings are consistent with results from studies conducted among SAC in the study area showing a decline in the prevalence of schistosomiasis attributed to drought conditions that dried most open water bodies during the period 2015–2017 [7, 34], they may not be comparable with results from PSAC in rural settings with different climate conditions.

## Conclusions

The prevalence of schistosomiasis in our study was low, suggesting that it may be possible to eliminate it among PSAC in the area provided vertical morbidity control interventions are integrated with community-wide health promotion strategies such as health education and the provision of clean water and basic sanitation. Our findings highlight the need for behavioural change interventions among caregivers of PSAC for schistosomiasis and soil-transmitted helminthiases control. The consideration of sociodemographic, water, sanitation and behavioural factors emerged as key in the success of soil-transmitted helminthiases and schistosomiasis control and elimination programmes. This is probably because the control of helminths largely depends on the progress towards addressing the root cause of infection beyond treating symptoms. Our study findings may add to the national health strategy for the inclusion of PSAC in NTD screening and treatment programmes. Findings also reveal contextual sociocultural gaps that may hinder existing control programmes from intensifying the control of schistosomiasis and soil-transmitted helminthiases and their possible elimination. The screening and treatment of PSAC could be integrated into existing child immunization programmes. Findings from the study recommended the inclusion of caregivers in schistosomiasis and soil-transmitted helminthiases control programmes.

## Additional file

Additional file 1: Multilingual abstracts in the five official working languages of the United Nations. (PDF 816 kb)

## Data Availability

Data supporting the findings of this study are available on reasonable request from the corresponding author [HGS]. The data are not publicly available because the study is part of a bigger project (TIBASA–http://tiba-partnership.org/) that is still ongoing.

## Abbreviations

AIDS: Acquired immune-deficiency syndrome
ANOVA: Analysis of Variance
*CI*: Confidence interval
ECD: Early childhood development
EPG: Eggs per gram
HIV: Human immunodeficiency virus
NTD: Neglected tropical disease
*OR*: Odds ratio
PSAC: Preschool aged children
SSA: Sub-Saharan Africa
STH: Soil-transmitted helminths
WHA: World Health Assembly
WHO: World Health Organization
