# Supplementary material for: Prevalence and risk factors of schistosomiasis and soil-transmitted helminthiases among preschool aged children (1–5 years) in rural KwaZulu-Natal, South Africa: a cross-sectional study
Source: Infect Dis Poverty. 2019 Jun 16;8:47. doi: 10.1186/s40249-019-0561-5 (PMC6571117; doi:10.1186/s40249-019-0561-5)
Supplement: Supplementary file 1 — Multilingual abstracts in the five official working languages of the United Nations. (PDF 816 kb) [file 40249_2019_561_MOESM1_ESM.pdf]

Translation of the abstract into the five official working languages of the United Nations

انتشار وعوامل الخطر من داء البلهارسيات والديدان المنقولة بالتربة بين الأطفال الذين تتراوح أعمارهم بين (1-5 سنوات) في المناطق الريفية كوازولو ناتال ، جنوب أفريقيا: دراسة مستعرضة

Hlengiwe Gwebu Sacolo, Moses Chimbari, Chester Kalinda

الخلفيه : على الرغم من الجهود المبذولة للسيطرة على الأمراض المدارية المهملة ، فإن البلهارسيا واالديدان المنقولة بالتربة لا تزال منتشرة على نطاق واسع في أفريقيا و جنوب الصحراء الكبرى. البيانات الحديثة تشير إلى ان هذه الإصابات منتشرة بين الأطفال وخاصه في المجتمعات الفقيرة. لذلك كان من الضرري ايجاد برامج للعلاج والتحكم الفعال في هذا الامرض . لذا كان الهدف من الدراسة هوتحديد انتشار وشدة وعوامل الخطر من البلهارسيا وعدوى بلهارسيا بين الأطفال في منطقة Ingwavuma و في منطقة uMkhanyakude في جنوب أفريقيا.

الطريقة: أجريت دراسة مقطعية بين 1143طفل الذين تتراوح أعمارهم بين 1 - 5 سنوات في 34 مركز من مراكز رياض الأطفال ومراكزتنمية الطفولة المبكرة وتم جمع البيانات حول عوامل الخطر باستخدام استبيان شبه منظم وتم استخدام اختبار Kruskal-Wallis لمقارنة الاختلافات في الاصابه وشدتها مع تقدم العمر و تم إجراء اختبار Pearson Chi-square والانحدار اللوجستي متعدد المتغيرلتقيم العلاقة بين حالة عدوى أطفال والاجتماعية والديموغرافية والأسريه والمياه ومتغيرات الصرف الصحي وممارسات النظافة في الاطفال ومقدمي الرعاية.

النتائج: لاحظنا انخفاض معدل انتشار مرض البلهارسيا بنسبه (1.0 ٪) و S. mansoni بنسبه (0.9 ٪) وعلي النقيض كان معدل انتشار أسكاريس لومبيكويدس (18.3٪) مرتفعًا مقارنةً بمشعرات تريكويرس (1.2٪) ، والديدان الخطافية (1.6٪) ، و التنيا (6.4٪). وكانت احتمالات الإصابة بمرض البلهارسيا أدنى بين الأطفال من سن (15-24 سنة) والذين كانوا تحت مقدمي الرعاية (0.1 ، 95 ٪,CI: 0.02-0.54)وأولئك الذين استخدموا ماء الصنبور للأغراض المنزلية (0.3 ، 95 ٪ , CI: 0.09 - 0.78) و كانت الإصابة بالبلهارسيا أعلى بين الأطفال الذين يستحمون في مياه النهر (17.4 ، 95٪, CI: 5.96 - 51.04.) ومن ناحية أخرى كانت عدوى STH أدنى بين الأطفال الذين لم يلعبوا في التربة (0.1 ، 95٪ ,CI: 0.51-0.28)، واستخدموا ماء الصنبور للأغراض المنزلية (0.5 ، 95٪ , CI: 0.27-0.80) و الأطفال تحت رعاية مقدمي الرعاية الأصغر سنًا (25 - 35 عامًا) (0.3 ، 95٪,CI: 0.10-0.75)وكان خطر الإصابة بعدوى STH الأعلى بين الأطفال الذين لم يغسلوا أيديهم بالصابون (3.5 ، 95٪,CI: 1.04-11.67) والأطفال الذين لم يتم تشذيب أظافرهم (3.6 ، 95٪, CI:1.75-7.26).

الاستنتاجات: النتائج تظهر انخفاض معدل انتشار وشدة العدوى من البلهارسيا وعدوى STH باستثناء A. lumbricoides بين الأطفال في سن ما قبل المدرسة. عوامل تنبؤ البلهارسيا وعدوى STH بين الأطفال في سن ما قبل المدرسة كانت مرتبطة بسن مقدمي الرعاية والحالة التعليمية والمياه والنظافة . وارتبطت عدوى STH حصريًا بعادات اللعب وغسل اليدين في الأطفال وأبرزت النتائج الحاجة إلى تضمين مقدمي الرعاية الأطفال في برامج الوقاية من البلهارسيا.

Translated from English version into Arabic by Ahmed Elrashedy

南非夸祖鲁-纳塔尔农村地区学龄前儿童（1–5 岁）血吸虫病和土源性蠕虫病的患病率及危险因素分析：一项横断面研究

Hlengiwe Gwebu Sacolo, Moses Chimbari, Chester Kalinda

摘要

引言：尽管我们一直在努力地控制被忽视的热带病，但是血吸虫病和土源性蠕虫病在撒哈拉以南非洲地区仍广泛流行。最近的数据表明，这些感染在贫困社区的学龄前儿童很普遍。了解血吸虫病和土源性蠕虫的感染模式以及在学龄前儿童中的流行情况对于其有效的治疗和控制至关重要。本研究的目的是确定南非 uMkhanyakude 区的 Ingwavuma 地区学龄前儿童的血吸虫病和土源性蠕虫病的患病率、感染强度和相关危险因素。

方法：在 34 所幼儿园和早期幼儿教育开发中心对 1143 名 1–5 岁的学龄前儿童进行横断面研究。采用半结构化问卷收集风险因素数据。采用 Kruskal-Wallis 检验比较不同年龄组感染强度的差异。采用皮尔逊卡方检验和多因素 logistic 回归分析学龄前儿童的感染状况、社会人口学特征、家庭、饮水和环境卫生变量与学龄前儿童和护理人员卫生习惯之间的关系。

结果：我们观察到埃及血吸虫(*Schistosoma haematobium*，1.0%)和曼氏血吸虫(*S. mansoni*，0.9%)的患病率较低。蛔虫的患病率(18.3%)高于鞭虫(1.2%)、钩虫(1.6%)和绦虫(6.4%)。年轻(15–24 岁)护理人员(0.1，95% *CI*: 0.02–0.54)和家庭使用自来水(0.3，95% *CI*: 0.09–0.78)的学龄前儿童中，血吸虫感染的概率最低。然而，在河水中洗澡的学龄前儿童血吸虫感染率较高(17.4，95% *CI*: 5.96–51.04)。另外，不在土壤中玩耍(0.1，95% *CI*: 0.51–0.28)、家庭使用自来水(0.5，95% *CI*: 0.27–0.80)和拥有年轻(25–35 岁)护理者(0.3，95% *CI:* 0.10–0.75)的学龄前儿童土源性蠕虫感染率最低。未使用肥皂洗手(3.5，95% *CI*: 1.04–11.67)和不修剪指甲(3.5，95% *CI*: 1.75–7.26)的学龄前儿童感染土源性蠕虫的风险最高。

结论：本研究结果表明，除了蛔虫外，学龄前儿童血吸虫病和土源性蠕虫病的患病率和感染强度均较低。儿童感染血吸虫和土源性蠕虫的危险因素可能与护理人员的年龄、受教育程度、饮水和卫生习惯有关。土源性蠕虫的感染与学龄前儿童游戏和洗手习惯有关。研究结果强调了将学龄前儿童的护理人员纳入血吸虫病和土源性蠕虫病防治计划的必要性。

Translated from English version into Chinese by Sheng-Lin Wang, edited by Pin Yang

CHINESE CENTER FOR DISEASE CONTROL AND PREVENTION
NATIONAL INSTITUTE OF PARASITIC DISEASES

Prévalence et facteurs de risque de la schistosomiase et des géohelminthiases chez les enfants d'âge préscolaire (de 1 à 5 ans) dans les régions rurales du KwaZulu-Natal, en Afrique du Sud : étude transversale

Hlengiwe Gwebu Sacolo, Moses Chimbari, Chester Kalinda

Résumé

Contexte : Malgré les efforts déployés dans la lutte contre les maladies tropicales négligées (MTN), la schistosomiase et les géohelminthiases restent largement répandues en Afrique subsaharienne. Des données récentes suggèrent une prévalence de ces parasitoses parmi les enfants d'âge préscolaire dans les communautés les plus pauvres. Afin de mettre en place des programmes de traitement et de lutte efficaces, il est indispensable de disposer de données factuelles sur les schémas d'infestation par la schistosomiase et les géohelminthiases et sur la prévalence de ces parasitoses parmi les enfants d'âge préscolaire (EAP). Le but de l'étude était de déterminer la prévalence, l'intensité et les facteurs de risque de la schistosomiase et des géohelminthiases parmi les enfants d'âge préscolaire de la région d'Ingwavuma, dans le district d'uMkhanyakude en Afrique du Sud.

Méthodes : Étude transversale menée chez les enfants âgés de 1 à 5 ans dans 34 écoles maternelles et centres de développement de la petite enfance (CDPE). Les données sur les facteurs de risque ont été recueillies à l'aide d'un questionnaire semi-fermé. Le test de Kruskal-Wallis a été utilisé pour évaluer les différences d'intensité de l'infestation selon l'âge. Le test de khi-deux de Pearson et une régression logistique multivariée ont été réalisés pour évaluer l'association entre le statut parasitaire des enfants d'âge préscolaire , les variables sociodémographiques, domestiques et relatives à l'eau et l'assainissement, ainsi que les pratiques d'hygiène des enfants et des personnes qui en avaient la charge.

Résultats : Nous avons observé une faible prévalence de *Schistosoma haematobium* (1,0 %) et de *S. mansoni* (0,9 %). La prévalence d'*Ascaris lumbricoides* (18,3 %) excédait celle de *Trichuris trichiura* (1,2 %), des ankylostomes (1,6 %) et des *Taenia* (6,4 %). La plus faible probabilité d'infestation par les schistosomes concernait les enfants d'âge préscolaire dont des personnes jeunes (15-24 ans) avaient la charge (0,1 ; *IC* à 95 % de 0,02 à 0,54) et ceux qui utilisaient l'eau du robinet pour les usages domestiques (0,3 ; *IC* à 95 % de 0,09 à 0,78). Cependant, l'infestation par les schistosomes était plus fréquente parmi les enfants d'âge préscolaire qui se baignaient dans les cours d'eau (17,4 ; *IC* à 95 % de 5,96 à 51,04). Par ailleurs, l'infestation par les géohelminthes était moins fréquente parmi les enfants d'âge scolaire qui ne jouaient pas dans la terre (0,1 ; *IC* à 95 % de 0,51 à 0,28), utilisaient l'eau du robinet pour les usages domestiques (0,5 ; *IC* à 95 % de 0,27 à

0,80) dont les adultes responsables étaient jeunes (25-35 ans) (0,3 ; *IC* à 95 % de 0,1 à 0,75). Le risque de géohelminthiase était maximal parmi les enfants d’âge scolaire qui ne se lavaient pas les mains avec du savon (3,5 ; *IC* à 95 % de 1,04 à 11,67) et ceux dont on ne coupait pas les ongles (3,6 ; *IC* à 95 % de 1,75 à 7,26).

Conclusions : Les résultats montrent une prévalence et une intensité d’infestation faibles pour la schistosomiase et les géohelminthiases, à l’exception d’*A. lumbricoides*, parmi les enfants d’âge scolaire. Les facteurs prédictifs de la schistosomiase et des géohelminthiases parmi les EAP étaient liés à l’âge, au niveau d’éducation et aux pratiques d’utilisation de l’eau et d’hygiène des personnes qui s’occupaient des enfants. L’infestation par les géohelminthes a été associée exclusivement aux habitudes de jeu et de lavage des mains des enfants. Les résultats ont mis en évidence la nécessité de faire participer les personnes ayant la charge d’enfants d’âge préscolaire aux programmes de prévention et de lutte contre la schistosomiase et les géohelminthiases.

Translated from English version into French by Marina Della Torre, proofread by Suzanne Assenat, through

TRANSLATORS
WITHOUT BORDERS

Распространенность и факторы риска шистосомоза и гельминтозов, передаваемых через почву, среди детей дошкольного возраста (1–5 лет) в сельской местности Квазулу-Натал, Южная Африка: перекрестное исследование

Хленгиве Гвебу Саколо, Моисей Чимбари, Честер Калинда

Аннотация

Справочная информация: Несмотря на усилия по борьбе с забытыми тропическими болезнями (ЗТБ), шистосомоз и гельминтозы, передаваемые через почву, по-прежнему широко распространены в странах Африки к югу от Сахары. Последние данные свидетельствуют о том, что эти инфекции распространены среди детей дошкольного возраста (ДДВ) в бедных сообществах. Данные о тенденциях и распространенности шистосомоза и гельминтозов, передаваемых через почву (ГПП), среди ДДВ очень важны для программ эффективного лечения и борьбы. Целью данного исследования было определение распространенности, активности и факторов риска шистосомоза и ГПП среди ДДВ в районе Ингвавума в округе Мханьякуде, Южная Африка.

Методы: перекрестное исследование проводилось среди 1 143 ДДВ в возрасте от 1 до 5 лет в 34 дошкольных учреждениях и центрах раннего развития детей (РРД) . Данные о факторах риска были собраны с использованием полуструктурированной анкеты. Для сравнения различий в активности инфекции в зависимости от возраста использовали тест Краскала-Уоллиса. Критерий согласия Пирсона-хи-квадрат и многомерная логистическая регрессия были выполнены для оценки связи между наличием инфекции у ДДВ, социально-демографическими, бытовыми, водными и санитарно-гигиеническими условиями и гигиеническими привычками ДДВ и лиц, осуществляющих за ними уход.

Результаты: Мы наблюдали низкую распространенность *Schistosoma haematobium* (1,0 %) и *S. mansoni* (0,9 %). Распространенность *аскарид* (18,3 %) была высокой по сравнению с *власоглавами* (1,2 %), анкилостомами (1,6 %) и *цепнями* (6,4 %). Вероятность заражения шистосомами была самой низкой среди ДДВ, за которыми наблюдали молодые (15–24 лет) воспитатели (0,1, 95% *ДИ*: 0,02–0,54), и тех, кто использовал водопроводную воду (0,3, 95% *ДИ*: 0,09–0,78) для бытовых целей. Однако шистосомоз встречался чаще среди ДДВ, которые моются в речной воде (17,4, 95 % *ДИ*: 5,96–51,04). С другой стороны, ГПП встречались реже всего среди ДДВ, которые не играли

на земле (0,1, 95 % *ДИ*: 0,51-0,28), использовали водопроводную воду для бытовых целей (0,5, 95% *ДИ*: 0,27-0,80) и уход за которыми осуществляли воспитатели молодого (25–35 лет) возраста (0,3, 95 % *ДИ*: 0,10–0,75). Риск заражения ГПП был самым высоким среди ДДВ, которые не мыли руки с мылом (3,5, 95 % *ДИ*: 1,04–11,67) и которым не обрезали ногти (3,6, 95 % *ДИ*: 1,75–7,26).
Выводы: Результаты показывают низкую распространенность и активность шистосомоза и ГПП, за исключением *аскаридоза* среди ДДВ. Факторы, прогнозирующие инфицирование шистосомозом и ГПП среди ДДВ, были связаны с возрастом воспитателей, уровнем образования, качеством воды и гигиеническими привычками. ГПП были связаны исключительно с играми и привычками мытья рук ДДВ. Результаты показали необходимость включения лиц, осуществляющих уход за дошкольниками, в программы профилактики и борьбы с шистосомозом и ГПП.

Translated from English version into Russian by Anna Kukharchuk, proofread by Veronika Demeshchik, through

La prevalencia y los factores de riesgo de la esquistosomiasis y la helmintiasis transmitida por el contacto con el suelo en niños en edad preescolar (1-5 años) en la zona rural de KwaZulu-Natal, Sudáfrica. Un estudio transversal

Hlengiwe Gwebu Sacolo, Moses Chimbari, Chester Kalinda

Resumen
Antecedentes: a pesar de los esfuerzos para controlar las enfermedades tropicales desatendidas (ETD), la esquistosomiasis y la helmintiasis transmitida por el contacto con el suelo aún prevalecen ampliamente en el África subsahariana. Datos recientes indican que estas infecciones prevalecen en los niños en edad preescolar (NEP) de comunidades pobres. Las pruebas sobre los patrones de infección y la prevalencia de la esquistosomiasis y de los helmintos transmitidos por el suelo (HTS) en los NEP son fundamentales para el tratamiento eficaz y los programas de control. El objetivo del estudio fue determinar la prevalencia, la intensidad y los factores de riesgo de la esquistosomiasis y de la infección por HTS en los NEP en la zona de Ingwavuma del distrito de uMkhanyakude, Sudáfrica.
Métodos: se realizó un estudio transversal en 1143 NEP de entre 1 y 5 años de edad en 34 escuelas y centros de desarrollo infantil temprano (DIT). Los datos sobre los factores de riesgo se recopilaron a partir de un cuestionario semiestructurado. Se utilizó una prueba de Kruskal-Wallis para comparar las diferencias en la intensidad de infección con la edad. Se realizaron una prueba de chi cuadrado de Pearson y una regresión logística multivariable para evaluar la relación entre el estado de infección de los NEP, las variables sociodemográfica, de hogar, agua y sanidad y las prácticas de higiene de los NEP y sus cuidadores.
Resultados: observamos una baja prevalencia de *Schistosoma haematobium* (1,0 %) y de *S. mansoni* (0,9 %). La prevalencia de *Ascaris lumbricoides* (18,3 %) fue alta en comparación con la de *Trichuris trichiura* (1,2 %), anquilostomas (1,6 %) y *Taenia* (6,4 %). La probabilidad de infección por esquistosomas fue menor en los NEP con cuidadores más jóvenes (15-24 años) (0,1, *IC* del 95 %: 0,02-0,54) y en aquellos que utilizaban agua del grifo (0,3, *IC* del 95 %: 0,09-0,78) para fines domésticos. Sin embargo, la infección por esquistosomas fue mayor en los NEP que se bañan en aguas fluviales (17,4, *IC* del 95 %: 5,96-51,04). Por otro lado, la infección por HTS fue menor en los NEP que no jugaban en el suelo (0,1, *IC* del 95 %: 0,51-0,28), utilizaban agua del grifo para fines domésticos (0,5, *IC* del 95 %: 0,27-0,80) y aquellos bajo el cuidado de personas más jóvenes (25-35 años) (0,3, *IC* del 95 %: 0,10-0,75). El mayor riesgo de infección por HTS fue en

los NEP que no se lavaban las manos con jabón (3,5, *IC* del 95 %: 1,04-11,67) y aquellos que no tenían las uñas cortas (3,6, *IC* del 95 %: 1,75-7,26).
Conclusiones: los resultados demuestran la baja prevalencia e intensidad de infección de la esquistosomiasis y de la infección por HTS, salvo *A. lumbricoides*, en los NEP. Los factores que predicen la esquistosomiasis y la infección por HTS en los NEP estuvieron relacionados con la edad de los cuidadores, el nivel educativo, el agua y las prácticas de higiene. La infección por HTS estuvo relacionada únicamente con los hábitos de juego y de lavarse las manos de los NEP. Los resultados destacaron la necesidad de incluir a los cuidadores de los NEP en los programas de prevención y de control de la esquistosomiasis y la HTS.

Translated from English version into Spanish by Mayra León, proofread by Rosario González, through

TRANSLATORS
WITHOUT BORDERS
